# The safety and potential efficacy of exosomes overexpressing CD24 (EXO-CD24) in mild-moderate COVID-19 related ARDS

**DOI:** 10.1186/s12931-024-02759-5

**Published:** 2024-04-01

**Authors:** Ioannis Grigoropoulos, Georgios Tsioulos, Artemis Kastrissianakis, Shiran Shapira, Orr Green, Vasiliki Rapti, Maria Tsakona, Thomas Konstantinos, Athina Savva, Dimitra Kavatha, Dimitrios Boumpas, Konstantinos Syrigos, Ioannis Xynogalas, Konstantinos Leontis, Vasileios Ntousopoulos, Vissaria Sakka, Zafeiris Sardelis, Andreas Fotiadis, Lamprini Vlassi, Chrysoula Kontogianni, Anastasia Levounets, Garyfalia Poulakou, Mina Gaga, Ronan MacLoughlin, Justin Stebbing, Nadir Arber, Anastasia Antoniadou, Sotirios Tsiodras

**Affiliations:** 1 4, Department of Internal Medicine, University General Hospital Attikon, Medical School, National and Kapodistrian University of Athens, 12462 Athens, Greece; 2 Integrated Cancer Prevention Center, Tel-Aviv Sourasky Medical Center, 6 Weizmann St., 6423906 Tel Aviv, Israel; 3 Department of Molecular Genetic and Biochemistry, Sackler Faculty of Medicine, Tel Aviv University, Tel Aviv, Israel; 4 3, Department of Internal Medicine, National and Kapodistrian University of Athens, Medical School, “Sotiria” General Hospital, 11527 Athens, Greece; 5 7, Respiratory Medicine Department “Sotiria” General Hospital, 11527 Athens, Greece; 6 Department of Surgery and Cancer, Anglia Ruskin University, London, UK; 7 Department of Life Sciences, ARU, Cambridge, UK; 8 R&D Science & Emerging Technologies, Aerogen Ltd., IDA Business Park, Dangan, Galway, Ireland; 9 School of Pharmacy and Biomolecular Sciences, Royal College of Surgeons, Dublin, Ireland; 10 School of Pharmacy and Pharmaceutical Sciences, Trinity College, Dublin, Ireland

**Keywords:** ARDS exosomes, CD24, EXO-CD24, Covid-19, Phase IIb

## Abstract

**Introduction:**

EXO-CD24 are exosomes genetically manipulated to over-express Cluster of Differentiation (CD) 24. It consists of two breakthrough technologies: CD24, the drug, as a novel immunomodulator that is smarter than steroids without any side effects, and exosomes as the ideal natural drug carrier.

**Methods:**

A randomized, single blind, dose-finding phase IIb trial in hospitalized patients with mild to moderate Coronavirus disease 2019 (COVID-19) related Acute Respiratory Distress Syndrome (ARDS) was carried out in two medical centers in Athens. Patients received either 10^9^ or 10^10^ exosome particles of EXO-CD24, daily, for five consecutive days and monitored for 28 days. Efficacy was assessed at day 7 among 91 patients who underwent randomization. The outcome was also compared in a post-hoc analysis with an income control group (n = 202) that fit the inclusion and exclusion criteria.

**Results:**

The mean age was 49.4 (± 13.2) years and 74.4% were male. By day 7, 83.7% showed improved respiratory signs and 64% had better oxygen saturation (SpO_2_) (p < 0.05). There were significant reductions in all inflammatory markers, most notably in C-reactive protein (CRP), lactate dehydrogenase (LDH), ferritin, fibrinogen and an array of cytokines. Conversely, levels of the anti-inflammatory cytokine Interleukin-10 (IL-10) were increased (p < 0.05). Of all the documented adverse events, none were considered treatment related. No drug-drug interactions were noted. Two patients succumbed to COVID-19. Post-hoc analysis revealed that EXO-CD24 patients exhibited greater improvements in clinical and laboratory outcomes compared to an observational income control group.

**Conclusions:**

EXO-CD24 presents a promising therapeutic approach for hyper-inflammatory state and in particular ARDS. Its unique combination of exosomes, as a drug carrier, and CD24, as an immunomodulator, coupled with inhalation administration, warrants further investigation in a larger, international, randomized, quadri-blind trial against a placebo.

**Supplementary Information:**

The online version contains supplementary material available at 10.1186/s12931-024-02759-5.

## Introduction

Acute Respiratory Distress Syndrome (ARDS) is a fatal clinical syndrome characterized by acute respiratory failure due to diffuse lung inflammation and edema. It is a life-threatening disease associated with high morbidity and mortality with three million new cases each year [1]. Approximately 20–40% of ARDS patients deteriorate rapidly within 24 h, due to a cytokine release syndrome (CRS) leading to respiratory failure, the need for assisted ventilation, and frequently death [2]. Currently, there is no medical therapy for ARDS. A lung protective strategy of mechanical ventilation remains the only disease-specific therapy shown to improve survival [3–5].

CD24, a small, heavily glycosylated glycosylphosphatidylinositol (GPI)-anchored protein, serves as a dominant innate immune checkpoint [6]. It crucially regulates cytokine and chemokine production by tight regulation of the Nuclear Factor-κΒ NF-ĸB pathway [7, 8].

Pattern recognition receptors (PRRs), such as NOD‐ or Toll-like receptors (NLRs or TLRs), retinoic acid-inducible gene-I (RIG-I)-like receptors (RLRs), C-type lectin receptors (CLRs), and absent in melanoma-2 (AIM2)-like receptors (ALRs)*,* recognize pathogen-associated molecular pattern (PAMPs)*,* derived from microorganisms, or components of injured cells called damage-associated molecular patterns (DAMPs), as endogenous danger signals, and trigger the activation of the innate immune system by stimulating the NF-ĸB pathway [9]. The NF-κB regulates multiple aspects of innate and adaptive immune functions and serves as a pivotal mediator of inflammatory responses. It regulates the expression of a number of pro-inflammatory genes, in particular cytokines and chemokines [8].

**Fig. 1:**
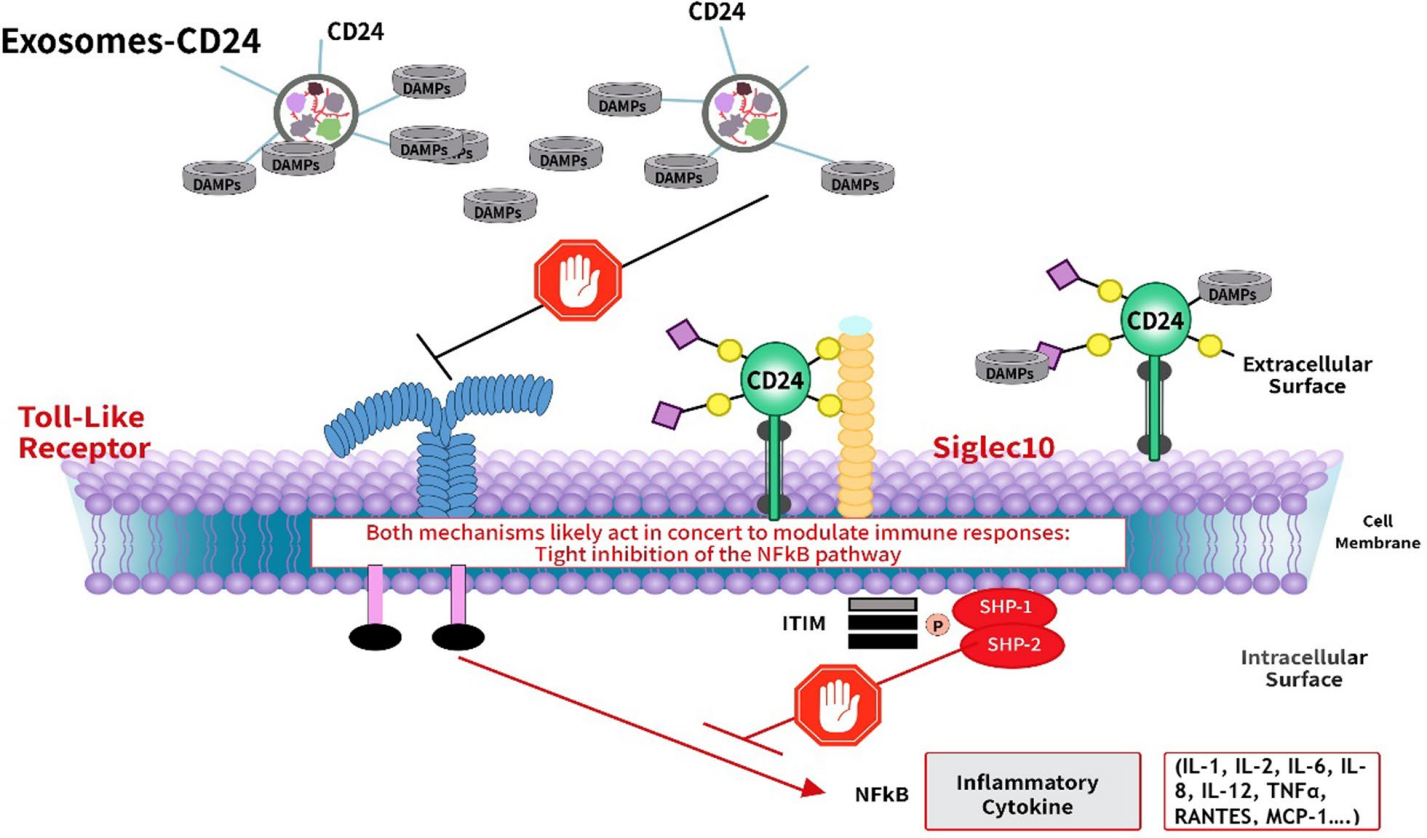
Negative regulation on the NF-ĸB pathway by the CD24-Siglec-10 axis. EXO-CD24 combine advantages of both exosomes as a novel drug delivery platform and CD24 as a potent immune checkpoint surveillance molecule. CD24 interacts with both DAMPs and Siglec 10. CD24’s link to DAMPs prevents them from binding to PRRs, such as the TLR, therefore inhibiting the NF-ĸB pathway that induces the cytokine storm. At the same time, the CD24-Siglec 10 axis negatively regulates the activity of NF-ĸB through ITIM domains associated with SHP-1. While CD24 interacts with DAMPs and Siglec 10, it does not affect immune recognition through PAMPs, thereby allowing the innate immune response to achieve viral clearance. *CD24* cluster of differentiation 24, *DAMPs* damage-associated molecular patterns, *ITIM* immunoreceptor tyrosine-based inhibition motif, *NF-κΒ* nuclear factor-κΒ, *PAMPs* pathogen-associated molecular patterns, *Siglec 10* sialic acid binding immunoglobulin-like lectin 10, *SHP-1* SRC homology 2-domain-containing protein tyrosine phosphatase 1, *TLR* toll-like receptor. Created with BioRender.com (accessed on 7 September 2022)

Sialylated CD24 serves as a negative signalling molecule to limit DAMP, but not PAMP- mediated inflammation. It selectively binds to DAMPs, preventing them from binding to PRRs, thereby inhibiting the NF-ĸB pathway [10]. Simultaneously, the CD24-Sialic acid binding Ig like lectin-10 (Siglec-10) axis, exert the opposite effect and down-regulate immune cell responses. CD24 as the major endogenous ligand for Siglec 10, negatively regulates the activity of NF-ĸB through immunoreceptor tyrosine-based inhibition motifs (ITIM) domains associated with Src Homology 2 Containing Protein Tyrosine Phosphatase-1 (SHP-1) (Fig. 1) [11].

Exosomes are intraluminal vesicles (30–200 nm) released by prokaryotic and eukaryotic cells [12–17]. Exosomes are the ideal and natural therapeutic drug carriers for several reasons, most notably for their low immunogenicity, stability, high bioavailability and high innate and acquired targetability [18–20]. The use of exosomes as therapeutic agents or as carriers of therapeutic agents, is a new and rapidly evolving area of research that has entered the clinical stage in recent years [21–25]. Exosomes are distributed throughout the respiratory system [26–28]. Nebulizers have already been shown as a promising exosome delivery device [29].

Our group has developed a unique platform of genetically engineered exosomes to over-express CD24, called EXO-CD24 [30].

In a phase Ib/IIa clinical study of 35 patients with mild-moderate COVID-19 related ARDS, EXO-CD24 showed promising efficacy and had a very favourable safety profile with no severe adverse events (SAEs) or even adverse events (AE) related to EXO-CD24 [31].

Eleven critically ill patients diagnosed with post-infection ARDS (ten with COVID-19 and one with an adenovirus infection) were administered EXO-CD24 in four medical centers across Israel. The administration of EXO-CD24 did not result in any recorded adverse events and demonstrated a promising efficacy [31].

In this randomized, single-blind, dose-finding phase IIb study, we aimed to confirm the safety and efficacy of EXO-CD24 in preventing clinical deterioration in patients with mild to moderate COVID-19 related ARDS.

## Materials and methods

### Clinical study

We conducted a randomized, single-blind, dose-finding, phase IIb clinical trial to assess the safety and potential efficacy of EXO-CD24. Patients with mild-moderate COVID-19 related ARDS, were recruited from two tertiary hospitals in Athens, Greece: University General Hospital ATTIKON and the SOTIRIA General Hospital of Chest Diseases.

Eligible patients were men and non-pregnant women 18 to 80 years old with a positive polymerase chain reaction (PCR) test for Severe Acute Respiratory Syndrome-Coronavirus-2 (SARS-CoV-2) within 30 days from screening, willing and able to sign an informed consent. Moderate or severe disease was defined by at least one clinical or radiologic finding (respiratory rate > 23/min and < 30/min, SpO_2_ at room air ≤ 94% and ≥ 90%, bilateral pulmonary infiltrates > 25% within 24–48 h of enrolment) and evidence of an exacerbated inflammatory process as indicated by laboratory parameters i.e., at least one of the following: CRP > 25 mg/L, ferritin > 500 ng/ml, lymphocytes < 800 cells/mm^3^ and D-dimers > 500 ng/ml.

Exclusion criteria included pregnancy or lactation, a current diagnosis of cancer, participation in any other interventional study within the last 30 days, previous complete or partial vaccination for SARS-CoV-2, and any concurrent illness that based on the judgment of the investigator could affect the interpretation or the results of the study (i.e., immunodeficiency).

Patients who were mechanically ventilated or were at-risk of mechanical ventilation and/or Intensive Care Unit (ICU) admission within 24 h were also excluded.

### Intervention

Patients were randomized to receive either 10^9^ or 10^10^ exosome particles per dose at a 1:1 ratio. Each dose was delivered using a standard compressed air-driven jet nebulizer at 5 L/minute for 4–5 min, for five consecutive days.

For patients in this cohort, the lung dose delivered by the jet nebulizer was estimated to be approximately 5% of the nominal dose, during spontaneous breathing [29].

Any concomitant medication deemed necessary by the treating physician was allowed in both treatment groups as standard of care, which was defined by national guidance issued by the Infectious Diseases Society of Greece and the ministry of health. It included remdesivir, dexamethasone and biologics like baricitinib or tocilizumab according to indications. The follow-up period was 28 days for safety and efficacy monitoring.

Randomization followed a stratified scheme using permuted random blocks.

Clinical assessments were performed on the initial day of EXO-CD24 administration, and continued on days 2–5, 7, and 28. These assessments included medical history reviews, physical examinations and documentation of any adverse events since the last assessment (refer to Protocol, p.55).

### Blood collection

Blood samples were obtained from all eligible participating patients at baseline and at several days during the EXO-CD24 treatment. Blood was collected into standard 9-ml collection tubes (Vacuette®, Greiner bio-one). All samples were collected and processed in an identical manner. Whole blood samples were centrifuged for 10 min at 4 °C at 3000 rpm. The plasma supernatant was then transferred to a new Corning® 15 ml tube and re-centrifuged at 1500×*g* for 10 min, brake 4, at 4 °C. After purification, plasma was divided into aliquots, preventing freeze–thaw cycles, and stored at − 80 °C until analysis.

Cytokine and chemokine levels were measured using glass slide multiplex Enzyme-Linked Immunosorbent Assay (ELISA) cytokine arrays according to the manufacturer’s guidelines (Quantibody®, RayBiotech). The Quantibody® array, is a multiplexed sandwich ELISA-based quantitative array platform, enables to accurately determine the concentration of multiple cytokines and chemokines simultaneously. A capture antibody is first bound to the glass surface. After incubation with the sample, the target cytokine is trapped on the solid surface. A second biotin-labeled detection antibody is then added, which can recognize a different epitope of the target cytokine. The cytokine-antibody-biotin complex can then be visualized through the addition of the streptavidin-conjugated Cy3 equivalent dye, using a laser scanner. In detail, one standard glass slide is divided into 16 wells of identical cytokine antibody arrays. Each antibody, together with the positive controls is arrayed in quadruplicate. For cytokine quantification, the array specific cytokine/chemokine standards, whose concentration has been predetermined, are provided to generate a standard curve for each cytokine/chemokine. By comparing signals from unknown samples to the standard curve, the cytokine/chemokine concentration in the samples was determined. The analysis is performed by using the Q-Analyzer. It is an array specific, Excel-based program.

### Outcomes

Primary safety endpoints, secondary efficacy endpoints, and exploratory endpoints are described below.

Primary safety endpoints were incidence of treatment-related serious adverse events, and incidence of all adverse events related to or unrelated to the study treatment. All serious adverse events were individually assessed by a medical expert team. Primary efficacy endpoints were:

1. The proportion of patients with respiratory rate < 23/min for at least 24 h on Day 7.
2. The proportion of patients with SpO2 saturation > 94%, on room air for at least 24 h on Day 7.
3. The proportion of patients with a decrease of 50% in either of the inflammatory markers (CRP or LDH or Fibrinogen or Ferritin or D-dimers) from baseline to Day 7.

Key secondary efficacy endpoints included:

1. The rate of categorical and absolute score improvement of COVID-19 status on Day 7 using any of the COVID-19 clinical severity ordinal scales (see Protocol), in each dose group and the total population.
2. Time to improvement in the ordinal scales measured from enrollment (Day 1) to the last study follow-up (Day 28).
3. Death rate on Day 28
4. Hospital discharge time calculated from the day of enrollment (Day 1) to discharge or last follow-up (Day 28), whichever occurs first.
5. The proportion of patients requiring admission to the ICU on Day 7.
6. The need for mechanical ventilation on Day 7.
7. The proportion of patients with improvement in respiratory rate (decrease > 2 breaths/min) and increase (> 2%) of SpO2 values from baseline to Day 7 (see protocol for full review of secondary efficacy endpoints).

An exploratory endpoint included inflammatory cytokine analysis and comparison before and after treatment with EXO-CD24.

### Post-hoc analysis

In a post-hoc analysis, patients included in the trial were compared with an incoming control cohort consisting of patients fulfilling the inclusion and exclusion criteria but declined participation in the clinical trial. We used their data as a comparison group under an already running prospective clinical infectious diseases protocol allowing the study physicians to collect data on biological parameters and outcome of infection (Approval by Ethics review board, University Hospital ATTIKON Oct 2016 and renew 2020: Δ’ΠΠΚ/Γ ΠΑΙΔ, ΕΒΔ 151/30-3-2020). All the control patients were recruited at the University General Hospital ATTIKON in Athens, Greece. The day of hospital admission was defined as baseline or Day 1 for the control cohort, and patients were observed prospectively. Outcomes of the post-hoc analysis were compared between the trial (EXO-CD24 group) and control cohort. Outcome measurements included:

1. The proportion of patients with SpO2 > 94% on room air, on Day 7.
2. The proportion of patients with a decrease of at least 50% in either one of the inflammatory indices [CRP, LDH, fibrinogen, ferritin, D-dimers, Neutrophil-to-Lymphocyte Ratio (NLR)] from baseline to Day 7.
3. The median improvement in National Institute of Allergy and Infectious Disease Ordinal Scale (NIAID-OS) from baseline to Day 7.
4. The proportion of patients with improvement of at least 1 point in the NIAID-OS from baseline to Day 7.
5. The ICU admission rate.
6. Median admission to discharge time in days.
7. The death rates.

### Statistical analysis

A sample size of 45 patients per group was calculated as adequate to detect a serious treatment-related adverse event rate of at least 5% with a probability of 90% (STATA 17.0). Safety assessments were performed on the intention-to-treat (ITT) population. The modified intention-to-treat (mITT) set included all patients randomized who received at least one dose of study treatment and met the study eligibility criteria retrospectively. The mITT cohort served as the principal data analysis set for the efficacy endpoints. The efficacy assessments were also performed on the per-protocol (PP) and ITT analysis sets to evaluate consistency of study results.

Continuous characteristics are presented as mean ± standard deviation (SD) or median values [interquartile range (IQR)], depending on the fulfillment of the normality assumption of their distribution (tested by Shapiro–Wilk and/or Kolmogorov–Smirnov tests). Categorical characteristics are presented as absolute numbers (N) and relative frequencies (%), based on the valid (non-missing) cases. No missing data imputation methodology was applied.

Safety and efficacy measures were compared among groups using Pearson Chi- square test or Fisher’s exact test as appropriate, for categorical variables or the independent samples t-test (or the Mann–Whitney U test as appropriate) in case of the continuous variables. Repeated measures analysis of variance was performed to analyze significance of changes of the examined variables/characteristics over time. The interaction between the treatment group and time was also examined. Additionally, Kaplan–Meier survival estimates with respective 95% Confidence Intervals (CIs) were calculated for all the time-to-event outcomes, while the log-rank test was performed for examining the difference between the two dosing groups. Simultaneously, Cox proportional hazards regression was also performed for the estimation of the Hazard Ratios (HRs) and their 95% CIs, regarding the difference between the two dosing groups. All statistical tests were two-tailed.

In the post-hoc analysis propensity score matching was used to balance baseline characteristics of EXO-CD24 and control groups. The variables selected for propensity score matching were age per 10 years, body mass index (BMI), cardiovascular disease or heart failure, diabetes mellitus and the NIAID-OS score on admission. Selection was based on known risk factors for severe COVID-19 pneumonia and differences in the baseline characteristics before matching. IBM-SPSS v24.0 and Stata v17.0 statistical software were used for the statistical analysis.

## Results

**Fig. 2:**
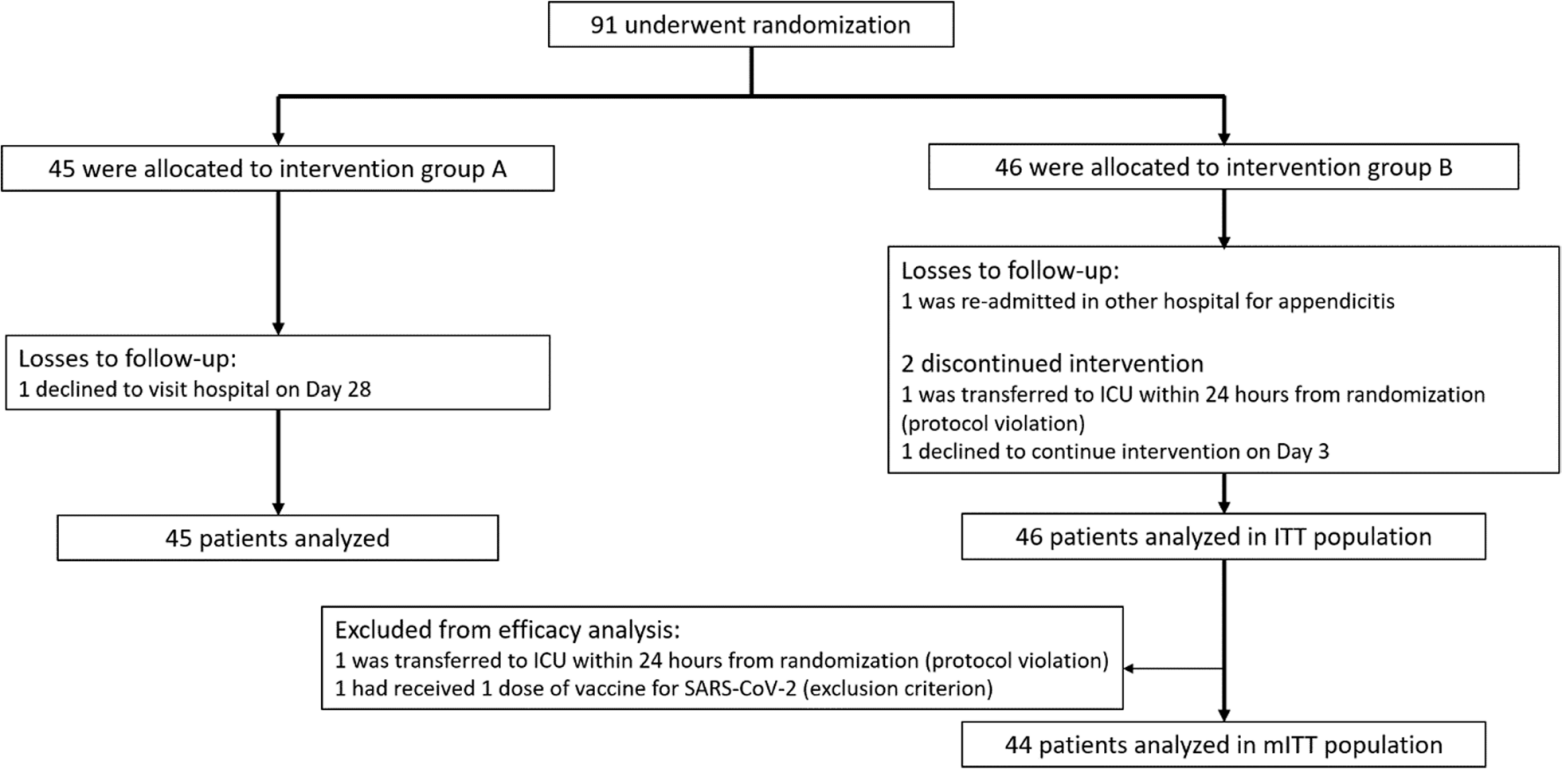
Flow chart*.* Group A: 10^9^ exosome particles per dose, group B: 10^10^ exosome particles per dose, *ICU* intensive care unit, *ITT* intention-to-treat, *mITT* modified intention-to-treat

Between June 9th 2021 and August 3rd, 2021, 91 patients underwent randomization: 45 patients were assigned to treatment group A (10^9^ exosome particles per dose) and 46 patients to treatment group B (10^10^ exosome particles per dose) (ITT population). One patient from group B declined receiving EXO-CD24 on days 3, 4 and 5, without withdrawing written informed consent. Therefore, the study coordinating team decided to proceed with an analysis including this patient.. Each treatment group had one patient lost to follow-up. Two patients from group B were excluded from the mITT and per protocol (PP) analyses, due to exclusion criteria violations. All other patients received their allocated treatment for five consecutive days and completed the predefined follow-up period. A breakdown of study participation is presented in Fig. 2.

Baseline demographic and clinical characteristics of the patients are presented in Table 1. The mean age was 49.4 years and 74.4% were male. The most common comorbidities were obesity [43.8%, defined by BMI ≥ 30 kg/m^2^], arterial hypertension (21.3%), dyslipidemia (19.1%) and diabetes mellitus (9.0%). The median duration from symptom onset to randomization was 8 days (7–11). The most frequent symptoms at baseline were cough (68.9%), fatigue (41.1%), shortness of breath (37.8%) and fever (34.4%) (see Additional file 1: Table S1). At baseline, 65 patients (73.0%) required supplemental oxygen via nasal cannula or venturi mask (NIAID-OS score 5) [25], while 18 (20.3%) needed high-flow oxygen devices (NIAID-OS score 6). The mean SpO_2_/Fraction of inspired oxygen (FiO_2_) ratio at baseline was 3.4 ± 1.0. Regarding concomitant medication, 75 (84.3%) patients received remdesivir, 75 (84.3%) received dexamethasone, 14 (15.9%) received baricitinib, and eight (9.0%) were administered tocilizumab. Baseline characteristics were evenly distributed between groups A and B (Table 1).

**Table 1:** Baseline demographic and clinical characteristics

	All patients (N = 89)	Group A^1^ (N = 45)	Group B^1^ (N = 44)	p-value
Age, years, mean (SD)	49.4 (13.2)	49.7 (14.2)	48.8 (12.3)	0.746
≤ 60, n (%)	69 (77.5)	35 (77.8)	34 (77.3)	0.954
> 60, n (%)	20 (22.5)	10 (22.2)	10 (22.7)	
Sex, n (%)				
Female	23 (25.8)	12 (26.7)	11 (25.0)	0.857
Male	66 (74.2)	33 (73.3)	33 (75.0)	
Ethnicity, n (%)				
Greek	82 (92.1)	44 (97.8)	38 (86.4)	0.058
Other	7 (7.9)	1 (2.2)	6 (13.6)	
BMI, kg/m^2^, mean (SD)	30 (5.7)	29.4 (4.5)	30.4 (6.6)	0.4
< 30, n (%)	50 (56.2)	26 (57.8)	24 (53.3)	0.759
≥ 30, n (%)	39 (43.8)	19 (42.2)	20 (46.7)	
Smoking status, n (%)				
Never	60 (67.4)	29 (64.4)	31 (70.5)	0.545
Former	19 (20.3)	11 (24.4)	8 (18.2)	0.714
Current	10 (11.2)	5 (11.1)	5 (11.4)	
Comorbidities, n (%)				
Hypertension	19 (21.3)	9 (20.0)	10 (22.7)	0.754
Dyslipidemia	17 (19.1)	7 (15.6)	10 (22.7)	0.39
Diabetes	8 (9.0)	4 (8.9)	4 (9.1)	> 0.999
CAD	3 (3.4)	3 (6.7)	0	0.242
Atrial fibrillation	3 (3.4)	2 (4.4)	1 (2.3)	> 0.999
COPD	2 (2.2)	2 (4.4)	0	0.494
CKD	1 (1.1)	1 (2.2)	0	> 0.999
Known immunosuppression	0	0	0	-
Score on NIAID-OS, n (%)				
4	6 (6.7)	3 (6.7)	3 (6.8)	
5	65 (73.0)	32 (71.1)	33 (75.0)	0.893
6	18 (20.3)	10 (22.2)	8 (18.2)	
Concomitant medication, n (%)				
Antibiotics	57 (64.0)	29 (64.4)	28 (63.6)	0.726
Remdesivir	75 (84.3)	38 (84.4)	37 (84.1)	> 0.999
Dexamethasone	75 (84.3)	39 (86.7)	36 (81.8)	0.56
Baricitinib	14 (15.9)	8 (18.2)	6 (13.6)	0.56
Tocilizumab	8 (9.0)	5 (11.1)	3 (6.8)	0.694
Anticoagulants				
Prophylactic	58 (66.7)	29 (65.9)	29 (67.4)	0.879
Intermediate	23 (26.4)	10 (22.7)	13 (30.2)	0.427
Therapeutic	8 (9.2)	6 (13.6)	2 (4.7)	0.147

*SD* standard deviation, *BMI* body mass index, *CAD* coronary artery disease, *COPD* chronic obstructive pulmonary disease, *CKD* chronic kidney disease, *NIAID-OS* National Institute of Allergy and Infectious Diseases ordinal scale ^1^Group A: 10^9^ exosome particles per dose, Group B: 10^10^ exosome particles per dose

### Primary efficacy endpoints

The proportion of patients with a respiratory rate < 23/min for at least 24 h on Day 7 was 83.7%. Sixty four percent of the patients had SpO2 saturation > 94% on room air for at least 24 h on Day 7, and 82.8% of the patients had a decrease of at least 50% in either of the inflammatory markers from baseline to Day 7. There was no significant difference between the two treatment groups in any of the primary efficacy endpoints (all p-values > 0.05), as shown in Table 2.

**Table 2:** Primary and secondary efficacy endpoints

	All patients (N = 86)	Group A (N = 43)	Group B (N = 43)	p-value
RR < 23/min for at least 24 h, on Day 7, n (%)	72 (83.7)	36 (83.7)	36 (83.7)	> *0.999*
SpO2 ≥ 94% on room air for at least 24 h, on Day 7, n (%)	55 (64.0)	25 (58.1)	30 (69.8)	*0.261*
Decrease by 50% in either of the inflammatory markers^2^ from baseline to Day 7, n (%)	72 (82.8)	35 (81.4)	37 (84.1)	*0.739*
NIAID-OS, on Day 7, n (%)				
1	5 (6.1)	2 (5.0)	3 (7.1)	
3	26 (31.7)	12 (30.0)	14 (33.3)	
4	17 (20.7)	8 (20.0)	9 (21.4)	*0.975*
5	21 (25.6)	11 (27.5)	10 (23.8)	
6	13 (15.9)	7 (17.5)	6 (14.4)	
Change in the NIAID-OS score from baseline to Day 7, median (IQR)	− 1 (− 2–0)	− 1 (− 2–0)	− 1 (− 2–0)	*0.537*
Time to improvement^3^ by at least 1 point in any of the COVID-19 clinical severity ordinal scales, median (95% CI)	6 (5.2–6.8)	6 (5.1–6.9)	4 (2.9–5.1)	*0.462*
Decrease > 2 breaths/min in RR from baseline to Day 7, n (%)	34 (39.5)	14 (32.5)	20 (46.5)	*0.120*
Increase > 2% of SpO2 values from baseline to Day 7, n (%)	28 (32.5)	14 (32.6)	14 (32.6)	*0.839*
Ready to be discharged^3^, days, median (95% CI)	7 (6–8)	7 (5.9–8.1)	7 (6.1–7.9)	*0.851*
ICU admission up to Day 7, n (%)	1 (1.2)	1 (2.3)	0	> *0.999*
Death outcome, n (%)	1 (1.2)	1 (2.3)	0	> *0.999*

*RR* respiratory rate, *SpO*_*2*_ blood oxygen saturation, *NIAID-OS* National Institute of Allergy and Infectious Diseases ordinal scale, *ICU* intensive care unit

^1^Group A: 10^9^ exosome particles per dose, Group B: 10^10^ exosome particles per dose

^2^Referring to: C-reactive protein or Lactate Dehydrogenase or Fibrinogen or Ferritin or D-dimers

^3^Kaplan–Meier survival estimates

### Secondary efficacy endpoints

**Fig. 3:**
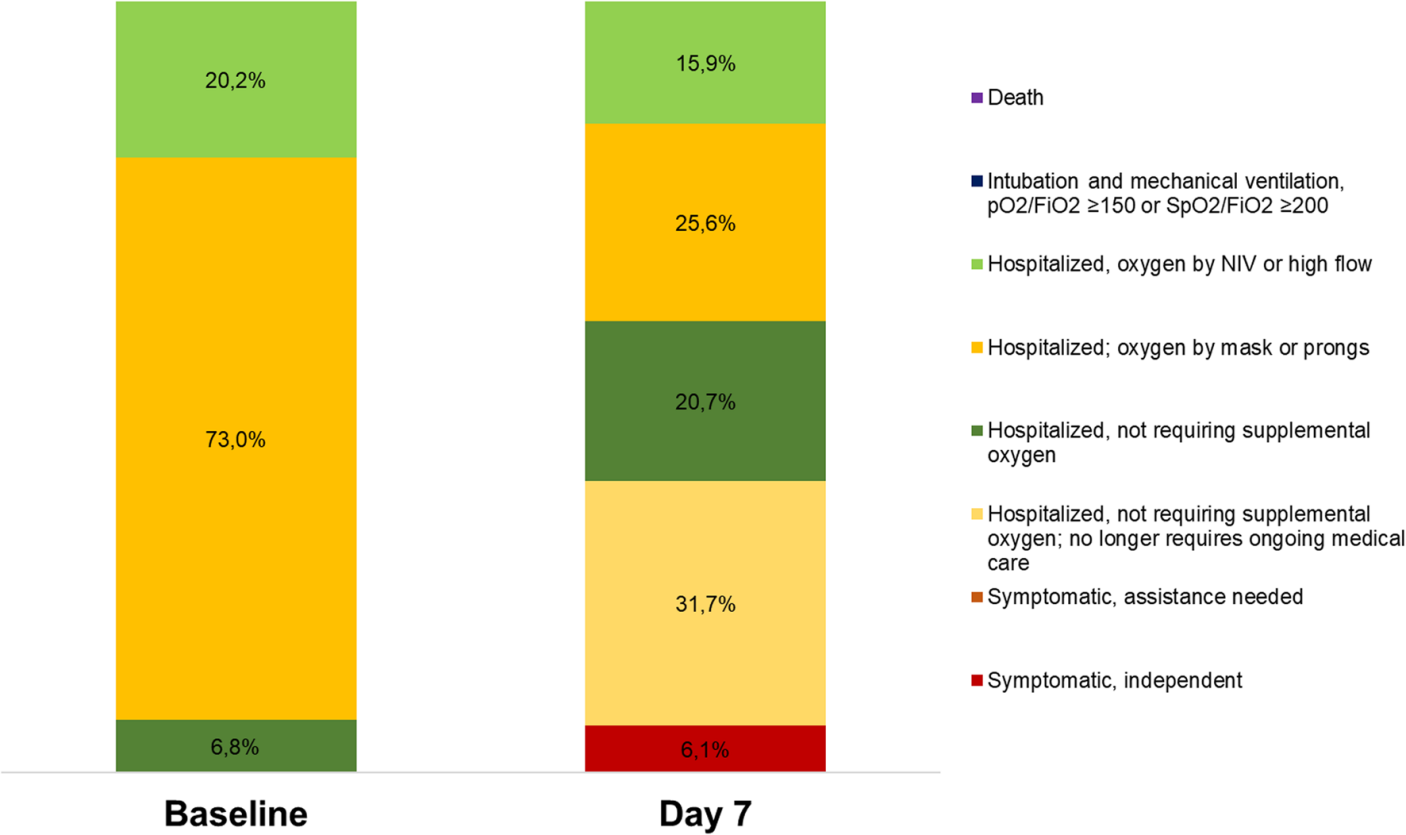
Distribution of the total sample of the patients according to their responses in the 8-point NIAID-OS scale at baseline and at Day 7*.* *NIAID-OS* National Institute of Allergy and Infectious Diseases ordinal scale, *NIV* non-invasive ventilation

**Fig. 4:**
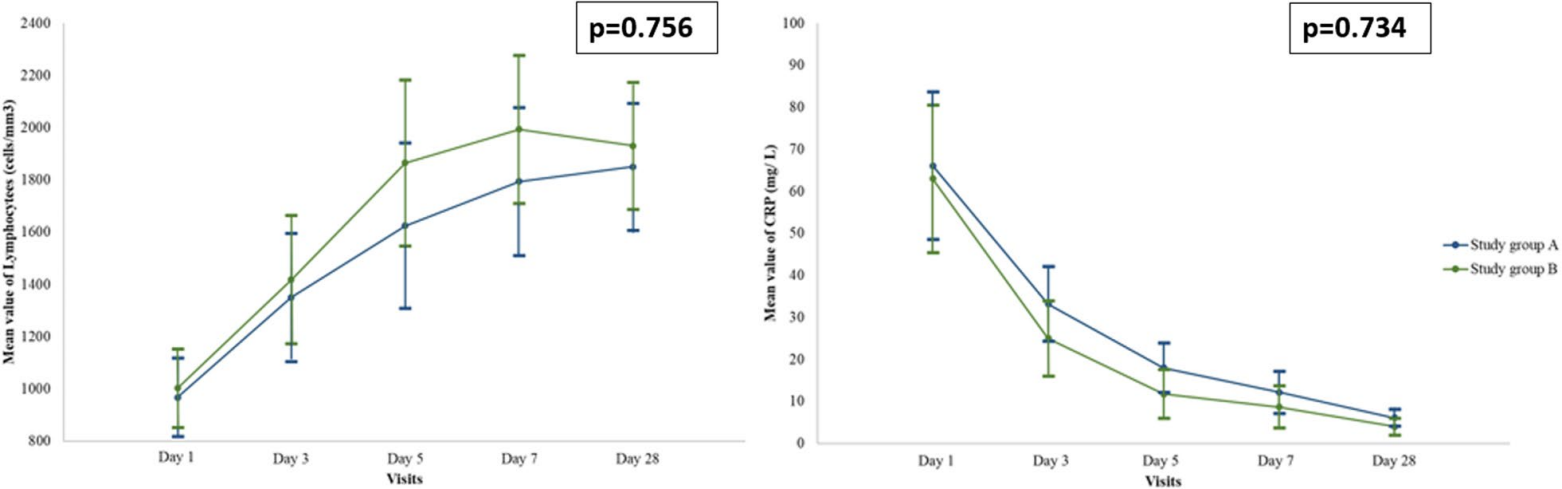
Mean values and change over time of absolute lymphocyte count and CRP, separately in each treatment group. Between-group differences were not statistically significant (p = 0.756 and p = 0.734 for lymphocyte count and CRP, respectively). Group A: 10^9^ exosome particles per dose, group B: 10^10^ exosome particles per dose

The distribution of the patients on the NIAID-OS at baseline and on Day 7 is shown in Fig. 3. Briefly, five (6.1%) patients were symptomatic-independent (NIAID-OS 2), 43 (52.4%) were hospitalized-not requiring supplemental oxygen (NIAID-OS 3 and 4), 21 (25.6%) were hospitalized receiving supplemental oxygen via mask or prongs (NIAID-OS 5) and 13 (15.6%) patients received supplemental oxygen via non-invasive ventilation or high-flow devices (NIAID-OS 6). The median change in NIAID-OS score from baseline to Day 7 was − 1 (− 2 to 0, p-value < 0.001), both in the total population and the two groups separately. Overall, 51 (62.2%) patients achieved an improvement of at least 1 point in the NIAID-OS from baseline to Day 7. The findings based on the World Health Organization (WHO) 7-point and WHO 10-point ordinal scales were similar (see Additional file 1: Table S2–S4). Median time to improvement by at least 1 point in any of the Covid-19 clinical severity ordinal scales was 6 days (95% CI: 5.1–6.9 days) in group A and 4 days (95% CI: 2.9–5.1 days) in group B. The between-treatment groups’ difference was not statistically significant (p-value = 0.462) (Fig. 4). Median hospital discharge time was 7 days (95% CI = 6–8 days) in the total population, with no statistically significant difference between the two groups (p-value of log-rank test = 0.851). Only one (1.2%) patient in the mITT cohort required invasive mechanical ventilation and ICU admission due to disease progression up to Day 7. Two (2.2%) patients died during the 28-day follow-up.

Mean SpO_2_/FiO_2_ ratio on Day 7 was 4.2 ± 0.8, resulting in an absolute increase of 0.8 from baseline to Day 7 (p-value = 0.002). The difference in increase of SpO_2_/FiO_2_ ratio was not statistically significant between groups A and B. The proportion of patients with improvement in respiratory rate (decrease > 2 breaths/min) from baseline to Day 7 was 39.5% in the total cohort, while a trend for higher rates of improvement was noted in group B (32.5% *vs* 46.5% for group A and B respectively, p = 0.12). Increase (> 2%) of SpO_2_ values from baseline to Day 7 was demonstrated in 32.5% of the patients with no statistically significant difference between groups (Table 2).

The mean values and change over time of absolute lymphocyte count and CRP are presented separately for each treatment group in Fig. 4. The between-group differences were not statistically significant (p = 0.756 and p = 0.734 for lymphocyte count and CRP, respectively).

### Primary safety outcomes

Adverse events (AE) are summarized in Table 3. Overall, 11 (12.1%) patients had at least one reported AE, while six patients (6.6%) had at least one reported serious adverse event (SAE) (Table 3). One patient experienced self-limited epistaxis. No allergic type of reaction or local adverse events, such as bronchospasm, cough or oral candidiasis were noted. None of the AEs/SAEs were considered treatment-related and no significant differences were noted between treatment groups (Table 3). Two (2.2%) patients of the ITT population died, after intubation and admission to the ICU. The first of these patients was an 80-year-old woman with multiple cardiovascular risk factors who was intubated due to cardiac arrest four days after the last dose of study treatment and was transferred to the ICU. The patient died because of bowel ischemia 15 days after ICU admission. The second patient was a 60-year-old male with hypertension and BMI of 38.6 kg/m^2^ who was intubated and transferred to the ICU within the first 24 h of enrollment due to severe respiratory failure and septic shock. Cardio-respiratory arrest occurred, and the patient died 29 days after admission to the hospital. Both incidents were evaluated by the independent primary medical team and were deemed to be unrelated to the study drug.

**Table 3:** Summary of serious/non-serious adverse events (AEs) by system organ class (SOC) in the intention-to-treat population (N = 91)

Serious/non-serious AEs by SOC	n (%)
Bradycardia	1 (1.1)
Cardio-respiratory arrest	2 (2.2)
Intestinal obstruction	1 (1.1)
Appendicitis	1 (1.1)
Septic shock	1 (1.1)
*Klebsiella spp.* bacteremia	1 (1.1)
Hyperkalemia	2 (2.2)
Postural dizziness	1 (1.1)
Epistaxis	1 (1.1)
Acute respiratory failure	2 (2.2)

None was assessed as treatment related

No drug-drug interactions were noted.

### Post-hoc analysis

A total of 202 consecutive patients were included in the control cohort. Seven patients were excluded due to insufficient clinical data. In comparison with the trial cohort, the patients in the control cohort were older (57.4 ± 12.0 vs. 49.2 ± 13.2, p < 0.001), had female predominance (41.1% vs. 25.8%, p = 0.013) and comorbidity differences e.g., hypertension (33.2% vs. 21.4%, p = 0.042), diabetes mellitus (18.3% vs. 9.0%, p = 0.043) and cardiovascular disease or heart failure (11.9% vs*.* 3.4%, p = 0.021). After propensity score matching, 72 patients in the EXO-CD24 cohort were compared with 70 patients in the control cohort. Baseline characteristics between the cohorts were similar (Table 4).

**Table 4:** Post-hoc analysis: baseline characteristics and outcomes after propensity score matching

	Controls (N = 70)	EXO-CD24^1^ (N = 72)	p-value
Age (mean ± SD), years	52.8 ± 12.0	52.3 ± 11.7	*p* = *0.766*
Male sex, n (%)	40 (57.1)	52 (72.2)	*p* = *0.06*
BMI ≥ 30 kg/m^2^, n (%)	29 (41.4)	31 (43.1)	*p* = *0.844*
Comorbidities, n (%)			
Current smoking	5 (7.4)	5 (6.9)	*p* = *0.925*
Hypertension	14 (20.0)	16 (22.2)	*p* = *0.746*
Dyslipidemia	11 (15.7)	14 (19.4)	*p* = *0.560*
Diabetes	7 (10.0)	7 (9.7)	*p* = *0.956*
CAD or HF	3 (4.3)	3 (4.2)	*p* = *0.972*
COPD or asthma	4 (5.7)	7 (9.7)	*p* = *0.372*
ESRD	0	0	*–*
Autoimmune diseases	1 (1.4)	5 (6.9)	*p* = *0.102*
NIAID-OS score, n (%)			*p* = *0.517*
4	0	1 (1.4)	
5	57 (81.4)	55 (76.4)	
6	13 (18.6)	16 (22.2)	
Concomitant medication, n (%)			
Antibiotics	48 (68.6)	49 (68.1)	*p* = *0.947*
Remdesivir	62 (88.6)	60 (83.3)	*p* = *0.370*
Dexamethasone	70 (100)	68 (94.4)	*p* = *0.045*
Biologics^2^	21 (30.0)	21 (29.2)	*p* = *0.913*
Anticoagulants			*p* = *0.237*
Prophylactic	52 (74.3)	46 (63.9)	
Intermediate	16 (22.9)	18 (25.0)	
Therapeutic	2 (2.9)	7 (9.7)	
SpO2 ≥ 94% on room air, on Day 7, n (%)	26 (37.1)	40 (58.0)	*p* = *0.014*
Decrease by 50% in either of the inflammatory markers^3^ from baseline to Day 7, n (%)	38 (54.3)	60 (83.3)	*p* < *0.001*
Improvement in NIAID-OS from baseline to Day 7, median (IQR)	0 (0–4)	1 (0–2)	*p* = *0.863*
Improvement in NIAID-OS of at least 1 point from baseline to Day 7, n (%)	34 (48.6)	44 (62.9)	*p* = *0.089*
Death, n (%)	0	1 (1.4)	*p* = *0.324*
ICU admission, n (%)	2 (2.9)	2 (2.8)	*p* = *0.977*
Admission to discharge in days, Median (IQR)	8.5 (6–11)	9 (7–14)	*p* = *0.544*

*EXO-CD24* exosomes overexpressing cluster of differentiation 24, *SD* standard deviation, *BMI* body mass index, *CAD* coronary artery disease, *HF* heart failure, *COPD* chronic obstructive pulmonary disease, *ESRD* end-stage renal disease, *NIAID-OS* National Institute of Allergy and Infectious Diseases ordinal scale, *SpO2* blood oxygen saturation, *ICU* intensive care unit

^1^EXO-CD24 represents patients from both Group A and Group B patients of the clinical trial after propensity score matching with the control cohort

^2^Biologics used as standard of care according to local guidelines were either baricitinib or tocilizumab

^3^Referring to: C-reactive protein or Lactate Dehydrogenase or Fibrinogen or Ferritin or D-dimers

The proportion of patients with SpO2 > 94% on room air on Day 7 was greater in the EXO-CD24 group compared to the control group (58% vs. 37.1%, p = 0.014). A decrease by at least 50% in either of the inflammatory markers, from baseline to Day 7, was higher in the EXO-CD24 group compared to the control group (83.3% vs. 54.3%, p < 0.001). All other outcomes of the post-hoc analysis did not differ significantly between the groups. The outcomes of the post-hoc analysis is shown in Table 4.

### Exploratory endpoint

#### Cytokines and chemokines array

**Fig. 5:**
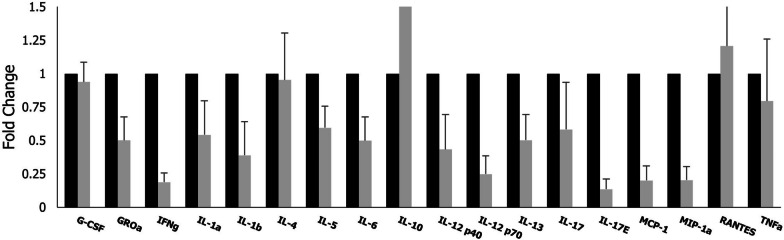
Cytokine analysis and comparison before and after treatment with EXO-CD24. Blood from all participating patients was collected at baseline and at Day 7 of the EXO-CD24 treatment. Cytokine and chemokine levels were analyzed using glass slide multiplex ELISA cytokine arrays according to the manufacturer instructions (Quantibody®, RayBiotech)

Biochemically, the serious effects are due to what is described as cytokine storm. Therefore, we performed a plasma cytokine array in the recruited patients to measure and verify the improvement in the inflammatory profile (Fig. 5). Majority of the cytokine and chemokine proteins were found to be up regulated in the infected individuals. Significant reductions of interferon γ, Interleukin (IL)-1a, IL-1b, IL-5, IL-6, IL-12, IL-13 and IL-17 were noted after treatment with the study medication (p < 0.01 for comparisons between baseline and Day 7 value). Conversely, the levels of anti-inflammatory cytokine IL-10, were significantly increased.

Τumor Necrosis Factor-α (TNF-α), IL-4, Granulocyte colony-stimulating factor (G-CSF) and Regulated upon Activation, Normal T Cell Expressed and Secreted (RANTES), did not change significantly before and after treatment.

## Discussion

The inhalation form of exosomes overexpressing CD24 (EXO-CD24), is a well-tolerated novel and effective treatment option in patients with mild-moderate COVID-19 related ARDS. EXO-CD24 as an add-on to standard of care therapy of ARDS patients has substantial beneficial clinical effects (oxygen saturation, respiratory rate), as well as improvements in biochemical parameters.

In this phase IIb, dose finding study, the safety of EXO-CD24 is confirmed. None of the SAEs and even of AEs were attributed to EXO-CD24. We did not observe any opportunistic infection during the follow-up of our patients despite their concurrent exposure to dexamethasone and biologics according to indications. Clinical outcome and laboratory parameters including cytokine and chemokine arrays, confirmed the promising efficacy of EXO-CD24 [32, 33].

In contrast to steroids, who shut down completely the entire immune system, EXO-CD24 has a distinct mechanism of action, as it allows immune discrimination between DAMPs, released from damaged or dying cells, and PAMPs, derived from pathogens such as bacteria and viruses [33]. The binding of CD24 to DAMPs traps them and prevents them from binding to PRRs, thereby inhibiting DAMP-activation of the NF-ĸB pathway. Additionally, CD24 binds to Siglec-10, resulting in activation of the inhibitory Siglec-10 signaling pathway, further inhibiting the activity of NF-ĸB pathway [34].

There are sheer number of cytokines and chemokines. There are many anti-cytokine and chemokine drugs, but targeting a specific one makes it nearly impossible to successfully overcome the entire cytokine storm [35]. In contrast, EXO-CD24 acts upstream of the cytokine storm, and broadly down regulates the expression of all cytokines and chemokines back to normal expression [33].

Aerosol-mediated EXO-CD24 administration has a significant clinical advantage in the treatment of critically ill patients, as it allows efficient delivery of the drug directly into the lungs [27].

EXO-CD24 was an add-on medication to standard of care regimens, thus allowing the extrapolation of the study findings to the real-life setting. Furthermore, the relatively short duration of patients’ enrollment allowed for no changes in standard of care regimens during that period.

The current work is limited by the study design which was primarily focused on the safety profile and dosage of EXO-CD24, while its efficacy was a secondary area of interest. This produces an inherent bias due to lack of a placebo group. Another limitation was the single-blind design of the study, which could have led to observer-expectation bias. Nevertheless, most of our study assessments were objective measures, which partially mitigates this concern. Additionally, time of initial diagnosis was not assessed in this trial, as the study treatment mainly aims at inflammatory response regulation and cytokine storm alleviation, which takes place later in the course of COVID-19. Since this was a clinical study, every effort was made to exclude other reasons for the clinical deterioration of patients such as co-infections, secondary infections or other conditions that could exacerbate the inflammatory milieu. Finally,the time of diagnosis in relation to treatment start could have affected the interpretation of the results. Nevertheless, diagnosis does not coincide with symptom onset and this interval is affected by several virological and immunological parameters that differ per affected patient. As the study treatment mainly aimed at inflammatory response regulation and cytokine storm alleviation, that usually takes place later in the course of COVID-19 (after the first 5–7 days) [2] and all patients presented at the trial triage team at the time of fulfilling clinical criteria for treatment, we believe that an interaction with result interpretation was minimal.

Our findings suggest that further investigation is warranted to better understand the effects of EXO-CD24 on ARDS of varying severity and etiologies beyond COVID-19. To this end, an international, quadri-blind, randomized trial of EXO-CD24 vs placebo is being currently underway.

EXO-CD24 may also serve as a breakthrough therapy for many other diseases with hyperinflammatory state and address an urgent, unmet need. For example, a proof of concept has been achieved in several animal models of abdominal and pulmonary sepsis, influenza, pulmonary fibrosis, asthma and COPD (Shapira et al., IJMS 2023). Since the cytokine storm is a common junction of ample of hyper-inflammatory diseases, targeting the cytokine storm with EXO-CD24 is the key to a successful cure for all these indications. [30, 32, 36–42].

EXO-CD24 can be lyophilized and could potentially be given by a spacer. This in turn could alleviate the need for nebulization devices and lower cost, treatment time and attention needed from medical personnel and provider [43].

EXO-CD24 is a novel, nebulized medication for hospitalized patients with ARDS-post COVID-19 infection. Our clinical trial suggests safety and potential efficacy of EXO-CD24 on clinical and laboratory parameters, as well as significant improvement of disease severity.

### Supplementary Information

**Additional file 1: Table S1**. Distribution of the patients according to the severity of the COVID-19 related symptoms at baseline. **Table S2.** Patients’ distribution according to their score in the 8-point NIAID-OS scale at baseline and at Day 7, both in total, as well as separately according to their treatment group. **Table S3.** Patients’ distribution according to their score in the 7-point WHO-OS scale at baseline and at Day 7, both in total, as well as separately according to their treatment group. **Table S4.** Patients’ distribution according to their score in the 10-point WHO-OS scale at baseline and at Day 7, both in total, as well as separately according to their treatment group.

## Data Availability

Study can be find at the clinicaltrials.gov website; ClinicalTrials.gov ID NCT04902183. The dataset supporting the conclusions of this article is available upon request from corresponding author.The data is available upon request from corresponding author.
