# Supplementary material for: The safety and potential efficacy of exosomes overexpressing CD24 (EXO-CD24) in mild-moderate COVID-19 related ARDS
Source: Respir Res. 2024 Apr 1;25:151. doi: 10.1186/s12931-024-02759-5 (PMC10983648; doi:10.1186/s12931-024-02759-5)
Supplement: Supplementary file 1 — Additional file 1: Table S1. Distribution of the patients according to the severity of the COVID-19 related symptoms at baseline. Table S2. Patients’ distribution according to their score in the 8-point NIAID-OS scale at baseline and at Day 7, both in total, as well as separately according to their treatment group. Table S3. Patients’ distribution according to their score in the 7-point WHO-OS scale at baseline and at Day 7, both in total, as well as separately according to their treatment group. Table S4. Patients’ distribution according to their score in the 10-point WHO-OS scale at baseline and at Day 7, both in total, as well as separately according to their treatment group. [file 12931_2024_2759_MOESM1_ESM.docx]

**Table S1. Distribution of the patients according to the severity of the COVID-19 related symptoms at baseline**

| **COVID-19 related symptoms- N (%)** | | **Total sample** | **Study Group A: CD24 exosomes at a dose of 10^9^ particles** | **Study Group B: CD24 exosomes at a dose of 10^10^ particles** | **p-value*** |
| --- | --- | --- | --- | --- | --- |
| **Stuffy or runny nose** | None | 78 (86.7) | 40 (88.9) | 38 (84.4) | 0.161 |
|  | Mild | 10 (11.1) | 3 (6.7) | 7 (15.6) |  |
|  | Moderate | 2 (2.2) | 2 (4.4) | 0 (0) |  |
|  | Severe | 0 (0) | 0 (0) | 0 (0) |  |
| **Sore throat** | None | 76 (84.4) | 39 (86.7) | 37 (82.2) | 0.496 |
|  | Mild | 12 (13.3) | 5 (11.1) | 7 (15.6) |  |
|  | Moderate | 1 (1.1) | 0 (0) | 1 (2.2) |  |
|  | Severe | 1 (1.1) | 1 (2.2) | 0 (0) |  |
| **Shortness of breath (difficulty breathing)** | None | 56 (62.2) | 29 (64.4) | 27 (60) | 0.326 |
|  | Mild | 23 (25.6) | 10 (22.2) | 13 (28.9) |  |
|  | Moderate | 9 (10) | 6 (13.3) | 3 (6.7) |  |
|  | Severe | 2 (2.2) | 0 (0) | 2 (4.4) |  |
| **Cough** | None | 28 (31.1) | 15 (33.3) | 13 (28.9) | 0.504 |
|  | Mild | 45 (50) | 24 (53.3) | 21 (46.7) |  |
|  | Moderate | 16 (17.8) | 6 (13.3) | 10 (22.2) |  |
|  | Severe | 1 (1.1) | 0 (0) | 1 (2.2) |  |
| **Low energy or tiredness** | None | 53 (58.9) | 28 (62.2) | 25 (55.6) | 0.693 |
|  | Mild | 26 (28.9) | 13 (28.9) | 13 (28.9) |  |
|  | Moderate | 7 (7.8) | 2 (4.4) | 5 (11.1) |  |
|  | Severe | 4 (4.4) | 2 (4.4) | 2 (4.4) |  |
| **Muscle or body aches** | None | 71 (78.9) | 39 (86.7) | 32 (71.1) | 0.132 |
|  | Mild | 13 (14.4) | 5 (11.1) | 8 (17.8) |  |
|  | Moderate | 6 (6.7) | 1 (2.2) | 5 (11.1) |  |
|  | Severe | 0 (0) | 0 (0) | 0 (0) |  |
| **Headache** | None | 68 (75.6) | 36 (80) | 32 (71.1) | 0.483 |
|  | Mild | 18 (20) | 8 (17.8) | 10 (22.2) |  |
|  | Moderate | 2 (2.2) | 1 (2.2) | 1 (2.2) |  |
|  | Severe | 2 (2.2) | 0 (0) | 2 (4.4) |  |
| **Chills or shivering** | None | 82 (91.1) | 40 (88.9) | 42 (93.3) | 0.095 |
|  | Mild | 6 (6.7) | 5 (11.1) | 1 (2.2) |  |
|  | Moderate | 2 (2.2) | 0 (0) | 2 (4.4) |  |
|  | Severe | 0 (0) | 0 (0) | 0 (0) |  |
| **Feeling hot or feverish** | None | 59 (65.6) | 30 (66.7) | 29 (64.4) | 0.797 |
|  | Mild | 20 (22.2) | 10 (22.2) | 10 (22.2) |  |
|  | Moderate | 10 (11.1) | 5 (11.1) | 5 (11.1) |  |
|  | Severe | 1 (1.1) | 0 (0) | 1 (2.2) |  |
| **Nausea (feeling like you wanted to throw up)** | None | 81 (90) | 41 (91.1) | 40 (88.9) | 0.541 |
|  | Mild | 7 (7.8) | 3 (6.7) | 4 (8.9) |  |
|  | Moderate | 1 (1.1) | 1 (2.2) | 0 (0) |  |
|  | Severe | 1 (1.1) | 0 (0) | 1 (2.2) |  |
| **How many times did you vomit in the last 24 hours?** | I did not vomit at all | 87 (96.7) | 42 (93.3) | 45 (100) | 0.242 |
|  | 1–2 times | 3 (3.3) | 3 (6.7) | 0 (0) |  |
|  | 3–4 times | 0 (0) | 0 (0) | 0 (0) |  |
|  | 5 or more times | 0 (0) | 0 (0) | 0 (0) |  |
| **How many times did you have diarrhoea (loose or watery stools) in the last 24 hours** | I did not have diarrhea at all | 78 (86.7) | 40 (88.9) | 38 (84.4) | 0.565 |
|  | 1–2 times | 11 (12.2) | 5 (11.1) | 6 (13.3) |  |
|  | 3–4 times | 0 (0) | 0 (0) | 0 (0) |  |
|  | 5 or more times | 1 (1.1) | 0 (0) | 1 (2.2) |  |
| **Rate your sense of smell in the last 24 hours** | My sense of smell is THE SAME AS usual | 61 (67.8) | 30 (66.7) | 31 (68.9) | 0.966 |
|  | My sense of smell is LESS THAN usual | 19 (21.1) | 10 (22.2) | 9 (20) |  |
|  | I have NO sense of smell | 10 (11.1) | 5 (11.1) | 5 (11.1) |  |
| **Rate your sense of taste in the last 24 hours** | My sense of taste is THE SAME AS usual | 62 (68.9) | 30 (66.7) | 32 (71.1) | 0.892 |
|  | My sense of taste is LESS THAN usual | 19 (21.1) | 10 (22.2) | 9 (20) |  |
|  | I have NO sense of taste | 9 (10) | 5 (11.1) | 4 (8.9) |  |

**P-value was given by the exact chi- square test regarding the comparison between the two treatment groups.*

# **Table S2. Patients’ distribution according to their score in the 8-point NIAID-OS scale at baseline and at Day 7, both in total, as well as separately according to their treatment group**

| **8-point NIAID- OS scale** | **Total sample** | **Study Group A: CD24 exosomes at a dose of 10^9^ particles** | **Study Group B: CD24 exosomes at a dose of 10^10^ particles** | **p-value** |
| --- | --- | --- | --- | --- |
| **At baseline** | **N= 89** | **N= 45** | **N= 44** |  |
| 4- Hospitalized, not requiring supplemental oxygen | 6 (6.7) | 3 (6.7) | 3 (6.8) | 0.893^1^ |
| 5- Hospitalized; oxygen by mask or prongs | 65 (73.0) | 32 (71.1) | 33 (75.0) |  |
| 6- Hospitalized, oxygen by NIV or high flow | 18 (20.3) | 10 (22.2) | 8 (18.2) |  |
| Median (IQR) | 5 (5-5) | 5 (5-5) | 5 (5-5) | 0.684^2^ |
| **At Day 7** | **N= 82** | **N= 40** | **N= 42** |  |
| 1-Symptomatic, independent | 5 (6.1) | 2 (5.0) | 3 (7.1) | 0.975^1^ |
| 3- Hospitalized, not requiring supplemental oxygen; no longer requires ongoing medical care | 26 (31.7) | 12 (30.0) | 14 (33.3) |  |
| 4- Hospitalized, not requiring supplemental oxygen | 17 (20.7) | 8 (20.0) | 9 (21.4) |  |
| 5- Hospitalized; oxygen by mask or prongs | 21 (25.6) | 11 (27.5) | 10 (23.8) |  |
| 6- Hospitalized, oxygen by NIV or high flow | 13 (15.9) | 7 (17.5) | 6 (14.4) |  |
| Median (IQR) | 4 (3-5) | 4 (3-5) | 4 (3-5) | 0.509^2^ |
| **Change in the 8-point NIAID- OS scale from baseline to Day 7** |  |  |  |  |
| Median (IQR) | -1 (-2, 0) | -1 (-2, 0) | -1 (-2, 0) | 0.537^2^ |
| *p-value* | *<0.001^3^* | *<0.001^3^* | *<0.001^3^* |  |

*Notes: 1p-value is based on the Pearson chi- square test; 2p-vaue is based on the Independent samples Mann- Whitney U test; 3p-value is based on the Wilcoxon signed rank test; IQR= Interquartile range expressed in the form 25th – 75th percentile.*

# **Table S3. Patients’ distribution according to their score in the 7-point WHO-OS scale at baseline and at Day 7, both in total, as well as separately according to their treatment group**

| **7-point WHO- OS scale** | **Total sample** | **Study Group A: CD24 exosomes at a dose of 10^9^ particles** | **Study Group B: CD24 exosomes at a dose of 10^10^ particles** | **p-value** |
| --- | --- | --- | --- | --- |
| **At baseline** | **N= 89** | **N= 45** | **N= 44** |  |
| 3- Hospitalized, not requiring supplemental oxygen | 5 (5.6) | 3 (6.7) | 2 (4.5) | 0.798^1^ |
| 4- Hospitalized; oxygen by mask or prongs | 64 (71.9) | 31 (68.9) | 33 (75.0) |  |
| 5- Hospitalized, oxygen by NIV or high flow | 20 (22.5) | 11 (24.4) | 9 (20.5) |  |
| Median (IQR) | 4 (4-4) | 4 (4-4.5) | 4 (4-4) | 0.826^2^ |
| **At Day 7** | **N= 82** | **N= 40** | **N= 42** |  |
| 1-Symptomatic, independent | 5 (6.1) | 2 (5.0) | 3 (7.1) | 0.922^1^ |
| 3- Hospitalized, not requiring supplemental oxygen | 43 (52.4) | 20 (50.0) | 23 (54.8) |  |
| 4- Hospitalized; oxygen by mask or prongs | 21 (25.6) | 11 (27.5) | 10 (23.8) |  |
| 5- Hospitalized, oxygen by NIV or high flow | 13 (15.9) | 7 (17.5) | 6 (14.3) |  |
| Median (IQR) | 3 (3-4) | 3 (3-4) | 3 (3-4) | 0.496^2^ |
| **Change in the 7-point WHO- OS scale from baseline to Day 7** |  |  |  |  |
| Median (IQR) | -1 (1, 0) | -1 (1, 0) | -1 (1, 0) | 0.470^2^ |
| *p-value* | *<0.001^3^* | *<0.001^3^* | *<0.001^3^* |  |

*Notes: 1p-value is based on the Pearson chi- square test; 2p-vaue is based on the Independent samples Mann- Whitney U test; 3p-value is based on the Wilcoxon signed rank test; IQR= Interquartile range expressed in the form 25th – 75th percentile.*

# **Table S4. Patients’ distribution according to their score in the 10-point WHO-OS scale at baseline and at Day 7, both in total, as well as separately according to their treatment group**

| **10-point WHO- OS scale** | **Total sample** | **Study Group A: CD24 exosomes at a dose of 10^9^ particles** | **Study Group B: CD24 exosomes at a dose of 10^10^ particles** | **p-value** |
| --- | --- | --- | --- | --- |
| **At baseline** | **N= 89** | **N= 45** | **N= 44** |  |
| 4- Hospitalized, not requiring supplemental oxygen | 6 (6.7) | 3 (6.7) | 3 (6.8) | 0.893^1^ |
| 5- Hospitalized; oxygen by mask or prongs | 65 (73) | 32 (71.1) | 33 (75) |  |
| 6- Hospitalized, oxygen by NIV or high flow | 18 (20.2) | 10 (22.2) | 8 (18.2) |  |
| Median (IQR) | 5 (5.5) | 5 (5.5) | 5 (5.5) | 0.684^2^ |
| **At Day 7** | **N= 87** | **N= 43** | **N= 44** |  |
| 1- Asymptomatic, viral RNA detected | 7 (8.5) | 5 (12.5) | 2 (4.8) | 0.330^1^ |
| 2-Symptomatic, independent | 3 (3.7) | 0 (0) | 3 (7.1) |  |
| 4- Hospitalized, not requiring supplemental oxygen | 43 (52.4) | 20 (50) | 23 (54.8) |  |
| 5- Hospitalized; oxygen by mask or prongs | 21 (25.6) | 11 (27.5) | 10 (23.8) |  |
| 6- Hospitalized, oxygen by NIV or high flow | 13 (15.9) | 7 (17.5) | 6 (14.3) |  |
| Median (IQR) | 4 (4,5) | 4 (4,5) | 4 (4,5) | 0.721^2^ |
| **Change in the 10-point WHO- OS scale from baseline to Day 7** |  |  |  |  |
| Median (IQR) | -1 (-1,0) | -1 (-1,0) | -1 (-1,0) | 0.745^2^ |
| *p-value* | *<0.001^3^* | *<0.001^3^* | *<0.001^3^* |  |

*Notes: 1p-value is based on the Pearson chi- square test; 2p-vaue is based on the Independent samples Mann- Whitney U test; 3p-value is based on the Wilcoxon signed rank test; IQR= Interquartile range expressed in the form 25th – 75th percentile.*
